# High-Resolution Movement EEG Classification

**DOI:** 10.1155/2007/54925

**Published:** 2008-01-15

**Authors:** Jakub Šťastný, Pavel Sovka

**Affiliations:** Biosignal Laboratory, Department of Circuit Theory, Faculty of Electrotechnical Engineering, Czech Technical University in Prague, Technická 2, Prague 16627, Czech Republic

## Abstract

The aim of the contribution is to analyze possibilities of high-resolution movement classification using human EEG. For this purpose, a database of the EEG recorded during right-thumb and little-finger fast flexion movements of the experimental subjects was created. The statistical analysis of the EEG was done on the subject's basis instead of the commonly used grand averaging. Statistically significant differences between the EEG accompanying movements of both fingers were found, extending the results of other so far published works. The classifier based on hidden Markov models was able to distinguish between movement and resting states (classification score of 94–100%), but it was unable to recognize the type of the movement. This is caused by the large fraction of other (nonmovement related) EEG activities in the recorded signals. A classification method based on advanced EEG signal denoising is being currently developed to overcome this problem.

## 1. INTRODUCTION

There are a great number of existing BCI prototypes all around the world. However, all of them suffer from one major drawback; the communication channel between a human brain and a computer is usually very slow working at a speed lower than 100 bits per minute. If we compare this communication channel with a standard keyboard computer interface allowing us to type texts at blazing speeds up to 1 kbit per minute, we can conclude that all these BCI devices are still not very suitable for the real computer control.

One possibility leading to higher data transfer lies in the recognition of more distinct brain states, which means transferring more bits per state via the communication channel (high-resolution EEG recognition), while keeping the average recognition score for the single states as high as possible. However, the currently existing systems recognize only few very different EEG phenomena (left/right- hand or finger movement [1–5], mental activities such as mental arithmetic, mental rotation, visual imagination [3, 4, 6, 7], conscious EEG rhythm control [8, 9], or event-related potentials [10–12], among others). Our research is targeted to the exploration of possibilities of the high-resolution movement recognition from the EEG signal. The movement-related EEG was selected because it is very natural to control anything with movement-related EEG as we usually control our surroundings in this way. It is well known that only imagination of the movement is sufficient [13, 14] to produce the desired brain activity pattern and last, but not the least, it is possible to change quickly the movement-related states of the brain compared , for example, to mental activities further increasing the interface transfer speed.

Our previous work showed that it is possible to distinguish right-shoulder and right-index finger movements easily from the EEG signal [15, 16] and classify the direction of the right-index finger movement on the basis of the EEG signal [17]. Movements performed at only one side of the body were used. This task is more complicated compared to differentiating only the left/right-hand movement or types of mental activities. The key requirement built into the classification system was to use changes in signal parameters rather than information stored in the difference of signal powers from different electrodes (extracted by means of appropriately defined spatial filters [18]), which lies in contrast with other existing systems. Encouraged by our previous result, we targeted our research to the development of a classification method which is able to recognize the single-finger movements from the EEG signal. Finger movements were chosen owing to the results of other works [19, 20].

The second important finding we learnt from our experiments is that the individualities of experimental subjects cause great differences between their recorded brain activities. These differences nowadays obstruct the possibility of the BCI generalization (the usage of the system trained on one subject for the movement classification of another subject). This led us to the conclusion that an individual approach to the EEG statistical analysis will be selected instead of the commonly used grand averaging (see, e.g., [20, 21]).

Last, but not least, the developed classification scheme [16] allows us to do a movement-related EEG classification without any need of subject training, which is a great advantage compared to other systems.

Our current work deals with the right-index and little-finger flexion movement-related EEG analysis and classification. This contribution is organized as follows. The general properties of the movement-related EEG are introduced in Section 2. Further, the EEG recording experiment is described. Section 4 section is devoted to a simple preliminary analysis of the recorded EEG proving the validity of our database. The core of our work is described in Sections 5 and 6. First we analyze the EEG in an individual way to find subtle differences between movements, then the classification system description and classification results are introduced. Finally, several conclusions and future steps are drawn.

## 2. GENERAL PROPERTIES OF THE MOVEMENT-RELATED EEG

We deal with movement-related changes of the EEG in the spectral domain in our work. The following characteristic phenomena are observed in the short-time EEG spectrum around the time of a movement (see [20, 21]).

**Figure 1:**
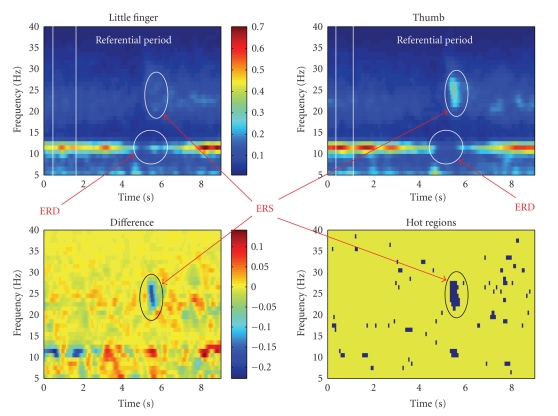
Example of the confidence intervals analysis (subject 1, electrode 4). The upper figures are the little-finger and thumb flexion PSD spectrograms. The time is biased to the movement onset—the movement was done in 0 second. Both figures share the same color scale. Lower left figure is the difference between both spectrograms. Some fluctuations can be seen at 11 Hz—μ-rhythm instabilities—and a difference in the βERS amplitudes is marked with a circle (positive difference = little-finger PSD which is at the given frequency and time instant larger than the thumb PSD). Finally, the lower right figure shows time-frequency combinations where the confidence intervals of both spectrograms are disjoint. Besides some random fluctuations, a clearly pronounced βERS region may be seen.

**Figure 2:**
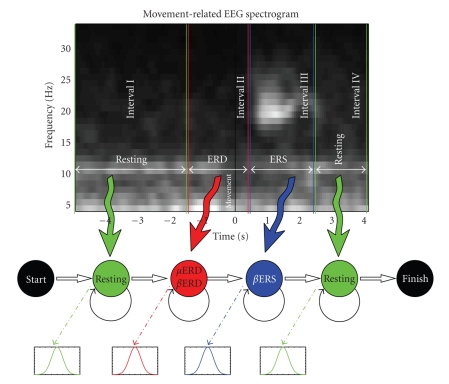
Used model architecture and its correspondence to the real EEG shape. The first and last emitting states model the resting period before and after the movement. The second emitting state holds the μand βERD characteristics, and the third one is related to the βERS.

μ *-rhythm event-related desynchronization* (μERD) starts about 1 second prior to the movement onset (see Figures 1 and 4 with marked ERDs and interval II in Figure 2). μERD is usually localized to the C3/CP3 and C4/CP4 scalp areas [14, 20, 22] and it exhibits a contralateral preponderance; usually we see two foci over both sensorimotor cortices. μERD allows to differentiate not only the side of the body performing the movement but slow and fast movements as well [23]. The desynchronization accompanies even the mere motor imagery and it is present in most normal adults' EEG.

β *-rhythm event-related desynchronization* (βERD) has a diffuse character over the scalp central area, and it is more widespread than βERS [14, 24]. βERD is at least partially coupled to the μERD showing the desynchronization at the frequency of the second harmonic component of the μ-rhythm [25]. Although there might be some components related to the βERS in the β-band during the ERD, there is no known evidence that they somehow allow to distinguish between different types of movement.

β *-rhythm event-related synchronization* (βERS) is displayed by central β rhythms as a rebound in the form of a phasic synchronization [24] after the movement. βERS represents a postmovement rise of power in the β-band; the phenomenon is located about 1 second after the movement onset (see Figures 1 and 4 with marked ERSs, and Figure 2 for interval III). βERS is larger over the contralateral hemisphere [24] and it is focused slightly anteriorly of the largest μERD. It is known that βERS allows to distinguish various types of movements such as wrist/finger flexion movements [20], index finger extension/flexion movements [26], or distal/proximal movements [21]. The βERS differences between extension/flexion movements and distal/proximal movements were successfully used for movement classification [16, 17].

## 3. EEG RECORDING

The following paragraphs describe the EEG recording and postprocessing procedures and provide basic characteristics of experimental subjects.

### 3.1. Experimental subjects

Eight subjects took part in the experiment—7 men and 1 woman with average age of 24.5 years (σ=3.59, see Table 1). None of them had a previous experience with such an experiment; all of them gave us an informed consent with the experiment and stated that they were healthy, without any known neurological problems, and were not under influence of any drug in the time of the experiment.

At first, we examined all the subjects on hand dominance. The [27] hand-dominance test consisting of three different tapping and drawing tasks was used. According to this test, four out of eight subjects were found to be right-handed, three as nonright-handed, and one as left-handed. The subjects with no significantly dominant hand were selected intentionally as the proposed system will work independently of the subject's handedness.

### 3.2. EEG recording setup

**Figure 3:**
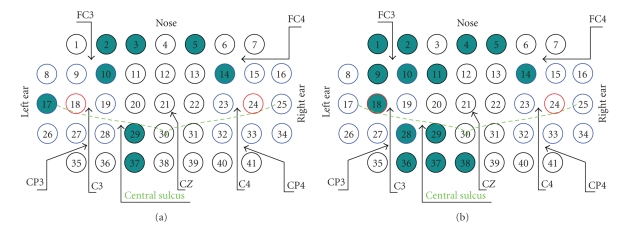
Localization of the electrodes allowing for the highest classification score and the real scalp electrode placement diagram. The 10–20 electrode positions C_3_, C_4_, and C_Z_ are denoted, and *central sulcus* is roughly localized. The electrode spacing is equidistant, 2.5 cm. Figures correspond to Table 5 (a) to the upper half: and (b) to the lower half; all the electrodes are shaded. Frontal locations (electrodes 1–16) correspond to the cases where the classifier distinguishes movement and resting EEG on the base of the βERS; classification on the parietal locations relies very likely more on the ERD. The best electrodes allowing to obtain the highest recognition score are placed contralaterally to the movement with the exception of electrode 5 (subject 8) and electrode 14 (subject 3 in Figure 3(a)) and electrodes 1 and 7 (in Figure 3(b)). All these subjects have a strong βERS present in the EEG and electrodes 5 and 14 are the anterior ones where the ERS is often present. The presence of the βERS thus allows the classifier to distinguish between resting and movement-related realizations here.

We used 41 unipolar scalp Ag/AgCl electrodes with 9 mm diameter placed symmetrically and equidistantly with 2.5 cm spacing [28] above both sensorimotor areas of the experimental subject (see Figure 3). Since the EEG changes in both time and space, the selection of appropriate EEG electrode representing movement is a crucial point for a successful classification of movements. As both movements are controlled primarily by the contralateral sensorimotor cortex, the most suitable electrodes are those overlying the contralateral sensorimotor hand area (electrode C3 and its surroundings) [14, 20]. The ground electrode was mounted on the nose, and impedances of all the electrodes were kept below 10 kΩ. The real exact positions of the scalp electrodes were measured with the help of the Isotrak II 3D scanner (manufactured by Polhemus, Colchester, Vermont, USA). In addition to the scalp electrodes, the following four bipolar channels were used: vertical and horizontal EOGs (electrodes placed horizontally and vertically along the subject's right eye), thumb EMG electrode placed on *thenar* recording the EMG of *musculus flexor pollicis brevis, m. opponens pollicis*, and *m. adductor pollicis*, and little-finger EMG electrode placed on *hypothenar* recording EMG of *m. flexor digiti minimi brevis* and *m. opponens digiti minimi*. The BrainScope EEG recording machine (manufactured by M&I, Prague, Czech Republic) was used for the EEG recording.

### 3.3. EEG recording procedure

The subject sat in a comfortable armchair in a silent and dim room with her/his right hand lying on the armrest in such a way so as she/he might freely perform the required thumb and little-finger movements. They were asked to keep their eyes closed and to avoid other movements than those asked for during the recording. Further, she/he was told to be as much relaxed as possible, but not to fall asleep. Before the recording was started, the subject was trained to perform the required movements properly.

The EEG was recorded in four blocks. The subject was performing the required self-paced voluntary movements during the first three blocks. The order and time between the movements were left at the subject's free will; no stimulation was used. This was to make the experimental procedure as much similar as possible to the real BCI usage. Two kinds of movements were performed during the recording—brisk flexions of the right thumb and the right little finger. Each of the three recording blocks contained about 30 movement; the blocks were separated by 10 minutes of rest.

During the fourth block, the resting EEG was recorded. We used this EEG as a referential one for false-movement detection rate estimation later on. The results of the experimental procedure were four blocks of about 15-minute-long EEG recordings per subject. The EEG was recorded with sampling rate of 256 Hz.

### 3.4. Data postprocessing

The first step was the temporal movement localization by means of visual inspection of EEG and EMG traces and by flagging the movement onsets. All the movements were found and tagged as either thumb or little finger. The resting period was tagged automatically by resting tag periodic insertion with 10-second period, with movement onset at the fifth second of the record.

Further we localized artifacts. Any movement or resting tag was changed into an artifact tag if any artifact was found in the 10-second-long epoch centred around the examined event. Also the EMG traces were checked and outliers were discarded.

The last step of the postprocessing was the Laplacian filtration with the 8-neighboring-electrodes Laplacian filter [29, 30]. Prior to the Laplacian filtration, the sequential sampling nature of our EEG machine was compensated by quadratic interpolation to improve the Laplacian output signal-to-noise ratio [31].

Since we wanted to perform a single-trial offline analysis and classification, we divided the EEG into 10-second-long epochs centered at the movement, with resting tags having the movement onset in the fifth second of the movement epoch. The resulting numbers of epochs for each of the subjects are listed in Table 1.

## 4. VERIFICATION OF THE NEW EEG DATABASE

The next step was to check whether our EEG was valid. We checked whether the movement-related phenomena in the recorded EEG were in compliance with previously published works [20, 21, 26] dealing with similar databases.

A standard method was used to extract the ERD and ERS parameters [21, 26, 29]. First, the average spectrograms giving the time development of the EEG power spectral density (PSD) for each subject, electrode and type of movement were computed (frequency resolution of 1 Hz, time resolution of 200 milliseconds, with Blackman window used; see Figure 1, e.g.). Averaging was done across all the realizations of the EEG belonging to one subject, movement, and electrode. Then the reference “resting” EEG power spectral density (PSD) (see Figure 1) was computed by averaging the PSDs belonging to time interval of 4.5−3.5 seconds before the movement onset. The average spectrograms were biased to this resting EEG giving a normalized course of the EEG PSD over time [29].

The most reactive frequencies for each of the subjects, electrodes, and type of movement were found in the μ- and β-bands—either the most attenuated one for ERD, or the most amplified one for ERS analysis. We performed a separate analysis for contralateral as well as ipsilateral sides of the scalp.

μ *-band ERD*: the following average frequency and ERD attenuation on the contralateral scalp side were obtained (average value ± one sigma estimation): * for little-finger flexion,* $f_{\text{avg}}=10.75\pm 0.67$ Hz, $\text{E}\text{R}{\text{D}}_{\text{avg}}=-83.0\pm 4.0\%$; *for thumb flexion,* $f_{\text{avg}}=10.88\pm 0.67$ Hz, $\text{E}\text{R}{\text{D}}_{\text{avg}}=-84.3\pm 3.4\%$. There were no significant differences apparent either between the ERD central frequencies or between the average ERD amplitudes for both fingers. The averaged frequencies and amplitudes computed for the ipsilateral scalp side were as follows: for *little-finger flexion,* $f_{\text{avg}}=10.75\pm 0.65$ Hz, $\text{E}\text{R}{\text{D}}_{\text{avg}}=-79.4\pm 3.2\%$; *for thumb flexion,* $f_{\text{avg}}=10.75\pm 0.65$ Hz, $\text{E}\text{R}{\text{D}}_{\text{avg}}=-82.5\pm 3.2\%$. Again, no significant differences were obtained. In addition to these averaged values, we analyzed the average ERD time courses across all the subjects. No significant differences were found either. Detailed results per subject are listed in Table 2.

Our results were compared to previously published results of experiments with similar EEG databases.

- Work [20] compares the μERD properties of right-index finger, little-finger, and wrist movements. The authors analyzed only the EEG recorded on C3 and C4 positions compared to our coverage of the whole sensorimotor scalp area. No differences between the little-finger and index-finger ERDs were found, which is in compliance with our findings.
- Works [22, 26] analyze μERD accompanying right-index finger brisk extensions and flexions. Although the authors chose different movements, we can at least compare the localization of the strongest ERD to our findings and see that we are in compliance with [26].
- In compliance with other works (e.g., [22]), the contralateral μERD was found to be stronger than the ipsilateral one in 6 out of 8 subjects.

β *-band ERS*: the results of our βERS analysis—the most reactive ERS components over both hemispheres—are given in Table 3. For all of the subjects but 3 contralaterally and for subjects 3 and 8 ipsilaterally, two distinct reactive bands (upper and lower) were found. The ERSs of subjects 2 and 4 β were very weak. We computed the ensemble average parameters for contralateral hemisphere (*little-finger flexion:* $f_{\text{avg}1}=22.8\pm 1.8$  Hz, $\text{E}\text{R}{\text{S}}_{\text{avg}1}=272\pm 75\%$, $f_{\text{avg}2}=18.0\pm 4.5$ Hz, $\text{E}\text{R}{\text{S}}_{\text{avg}2}=86\pm 28\%$; *thumb flexion:* $f_{\text{avg}1}=21.8\pm 1.8$ Hz, $\text{E}\text{R}{\text{S}}_{\text{avg}1}=223\pm 53\%$, $f_{\text{avg}2}=13.8\pm 4.5$ Hz, $\text{E}\text{R}{\text{S}}_{\text{avg}2}=104\pm 45\%$) as well as ipsilateral hemisphere (*little-finger flexion:* $f_{\text{avg}}=22.9\pm 2.7$ Hz, $\text{E}\text{R}{\text{S}}_{\text{avg}}=156\pm 38\%$; *thumb flexion:* $f_{\text{avg}}=21.8\pm 1.8$ Hz, $\text{E}\text{R}{\text{S}}_{\text{avg}}=188\pm 58\%$). It is obvious that there are no significant differences between either movement parameters. Further, in the grand average courses, no differences were found either.

Compared to other works the following can be concluded that

- work [20] did not find any significant difference between right-index finger and little-finger flexion βERSs (this is in compliance with our findings),
- work [26] analyzes the βERS accompanying the right-index finger extension and flexion movement; the strongest ERS is localized 2.5 cm anteriorly and about 5–7.5 cm left from the Cz position; we found the strongest thumb and little-finger ERS locations roughly in the same area.

The results listed above clearly show that the database is usable for our experiments and contains reliable movement-related EEG. The analysis results of the most reactive EEG frequency components are in compliance with previously published works with similar EEG recordings. No systematic differences between the EEGs of both movements are apparent. Results summarized in Tables 2 and 3 show no common relation between the βERS and μERD of the thumb and little-finger flexions (e.g., ERS of the thumb is *not* always stronger than ERS of the little finger).

## 5. INDIVIDUAL EEG ANALYSIS

Our previous experiments with the EEG signal classification clearly showed that there are large differences in the EEG signals of different subjects. Although we usually observed the same phenomena in the EEG recordings of different subjects, the individual parameters were different. This observation led us to the conclusion that the standard approach to the movement-related EEG patterns analysis via grand averaging the ERS and ERD over all subjects would not be suitable for finding subtle differences between both movement-related types of the EEG. The grand averaging wipes out any individual EEG differences between both movements which are not systematic (i.e., the same trend occurred across all the subjects). That is why we did a deep statistical analysis of individual EEG patterns to find any statistically significant phenomena in the EEG allowing us to recognize the finger which performed the movement.

### 5.1. Method

For each of the subjects (subject s=1,…,8), electrodes (electrode e=1,…,41), types of the EEG (type *m = {littel finger, thumb}*), and realizations (realization r=1,…,R(s,m); R(s,m) is the number of realizations available for the given subject s and type of the EEG m), a spectrogram ${𝒮}_{s,e,m,r}[f,t]$ was computed. The frequency resolution was 1 Hz (f=0,…,128; frequency in Hz) and time resolution was 0.125 second (t=0,…,72; time in 1-second segments with 0.875-second overlap [16]). The spectrograms described the time development of the short-time EEG power spectra. Next, we computed the average spectrogram 𝒮^s,e,m for the given subject s, electrode e, and type of the EEG m by averaging ${𝒮}_{s,e,m,r}$ across all available realizations r=1,…,R(s,m) (see Figure 2). 𝒮^s,e,m[f,t] describes the time development of the short-time EEG power spectral density (PSD). No referencing to the resting EEG referential period was applied here because we wanted to analyze exactly the same spectra as those which would be used for the classification later on.

As the PSD is $\chi^{2}$-distributed [32] with degrees of freedom equal to two times the number of realizations (2R(s,e,m)), we can simply find the 95% confidence-level interval as

(1) 2R(s,e,m)𝒮^s,e,mχ2R(s,e,m),0.0252≤𝒮^s,e,m≤2R(s,e,m)𝒮^s,e,mχ2R(s,e,m),0.9752.

We computed these confidence intervals for all the spectrograms and found out where there were disjoint for both types of movements. These areas were marked as “hot,” and thus we devoted our attention to them (see Figure 1).

As the processed spectrograms were non-Gaussian and since we needed to analyze the single frequency bins of the spectrum, we applied Kruskal-Wallis nonparametric test (KWT) of equal population means to the computed spectrograms in the “hot” areas instead of the commonly used ANOVA which requires Gaussianity.

The KWT was applied to the precomputed PSD spectrograms giving us the confidence that the average PSD values really differed between subjects. The confidence was thresholded at the 95% confidence level, and regions in which the average values differed were found. Then we passed through all the subjects across all the electrodes by hand looking for these regions, and we tried to summarize and systematize this rather large amount of data. The results of this analysis are the subjects of the following paragraphs.

The results discussed below need not necessarily be in accordance with the results listed in Tables 2 and 3 which were achieved by an approach commonly used by neurologists relying on searching for the most reactive ERD and ERS components and comparing them. Instead, here we tried to locate as many statistically significant individual differences between both movements as possible.

### 5.2. β-band ERS

The β-rhythm ERS analysis gave us the most valuable results. Our conclusions for the given experimental subjects are summarized in Table 4; it may be clearly seen that we did not obtain any systematical differences between the PSDs of both movements' EEGs. For subjects 1, 3, and 6, the ERS of the thumb flexion was stronger; for subjects 5 and 8, the little-finger flexion ERS was more pronounced; subject 7 showed both cases depending on the scalp location, and no significant differences were found for subjects 2 and 4. Note that the electrodes at which the ERSs are stronger for the thumb flexion are located more anteriorly compared to the electrodes with stronger little-finger ERSs (except subject 5, electrode 1). This is in compliance with the more lateral and anterior representation of the thumb compared to the more medial localization of the little finger in the M1 and S1 areas [19]. Interestingly, this trend did not appear in the most reactive ERS analysis (see Table 3), where the strongest ERS courses for both fingers were often found at the same electrode.

In the later classification experiments, we reached a significant level of the movement-resting EEG discrimination on some of the electrodes mentioned in Table 4 (marked with boldface). This fact implies that there must be strong statistically significant differences between the resting EEGs and movement-related EEG realizations as well. Our examination here shows statistically significant differences between both movements. All these findings imply that it should be possible to discriminate the movements after some suitable EEG postprocessing.

Some more differences were found in addition to these listed above, but they marked only changes in duration or bandwidth of the ERS between both movements. We did not list them here because they are outside the scope of this paper.

### 5.3. μ-band ERD

Although the μERD parameters are believed not to be dependent on the type of the movements [20], we analyzed the μERD behavior with the test mentioned above. We discovered the following phenomena:

- for subject 4, the little-finger μERD around the movement onset was found to be stronger than the thumb μERD at some of the locations (electrodes 20, 21, 25, 27, 29),
- finally we found some differences in the length of μERD of both movements.

### 5.4. β-band ERD

We also briefly examined ERD in β-band in order not to neglect anything which might be helpful or interesting. We found significant differences in the βERDs with one subject—subject no. 1, electrodes 9, 10, and 26, where the thumb-related βERD was significantly stronger than the little-finger flexion-related one. The frequency of the most reactive βERD component was 25–26 Hz. The βERD was observed in the same band as the βERS. The βERD frequency band did not contain the frequency of the μERD second harmonic component, and thus it was not related to the ongoing μERD.

## 6. CLASSIFICATION

The next step was to test the possibility of a single-trial offline classification. The following paragraphs describe the classification paradigm, parameterization, and results.

We intentionally always used only one electrode for the EEG classification. Our target was to squeeze as much information as possible from only one signal source, without utilizing any information stored in differences between signals from different electrodes.

### 6.1. Classifier

The used classification system is based on Hidden Markov models (HMM) [16, 33]. The HMMs —although nearly not used for EEG classification—have several advantages:

*utilization of the context information:* the system uses the temporary context of the EEG to improve the classification score,

*physiological compatibility:* the selected model architecture matches the underlying physiological process, it is even possible to segment the EEG with the help of the HMM classifier [16, 34],

*ease of the interpretation:* it is quite simple to interpret the contents of the trained model. This is a big advantage compared to, for example, some kinds of neural networks, where the implementation of the trained system is not so straightforward,

*ability to model the EEG:* we are able to generate synthetic realizations of the EEG for testing of various algorithms.

The used models have the left-to-right, no skips architecture which captures the sequence of the movement-related EEG phases (see Figure 2) with 4 emitting states modelling the four significant phases of movement-related EEG [16, 21] (resting EEG, desynchronization, post-movement synchronization, resting EEG) generating p-dimensional Gaussian random processes (p is equal to the number of used EEG features, per the Parameterization paragraph below) with diagonal covariance matrices. The used classification system was the same as in our other EEG BCI works [16, 34, 35] built around the Hidden Markov Toolkit [36]. The classification experiment consisted of the following steps performed for all the subjects, electrodes, and types of parameterization:

1. EEG was parameterized with a selected algorithm,
2. the randomization procedure was applied to mitigate the effect of the small training and testing set (only ≈100 realizations per movement, person, and electrode). Each classification experiment was run for 16 times with different (and random) division of EEG realizations between the disjunctive training (75% of realizations) and testing (25% of realizations) sets. The number of runs was selected to get 99% probability that any of the realizations is used for testing. This helps us to get reliable results independent on the concrete selected training and testing EEG realizations [34],
3. models were trained (initialization followed by Baum-Welch reestimation) on the training set,
4. classification accuracy was tested,
5. the average classification scores were computed for all the EEG types across the 16 performed experiments.

### 6.2. EEG parameterization

Our previous results [15] showed that the best results are reachable either with a pure FFT linear spectrum or with AR model coefficients combined with Δ parameters. The Δ parameters (although not used with EEG signal processing) are able to improve the classification score significantly [15]. This is a result of emphasizing the movement-related spectral changes which allows the classifier to better capture the underlying signal statistics. In all cases, we extracted the features from a sliding window of 1 sec length; step of the window was chosen as 200 ms [16]. We utilized the following parameterizations:

*linear spectrum:* FFT amplitude spectrum covering 5–40 Hz band with spectral resolution of 1 Hz. The k-th feature vector consisted of 36 parameters $F_{k}=(f_{k}[1],\ldots ,f_{k}[36])$ where k is the time index,

*linear spectrum +* Δ *coefficients:* additional 36 $\Delta f_{k}[i]$ coefficients were added to the already computed linear spectrum. The following polynomial approximation of the first derivative common in speech processing was used [36]

(2) $$\Delta f_{k}[i]=\frac{\sum_{l=1}^{3}l(f_{k+l}[i]-f_{k-l}[i])}{2\sum_{l=1}^{3}l^{2}},$$

AR coefficients: 8th order AR model coefficients [15] were used; the feature vector had 8 coefficients here. The EEG was decimated by factor 2 before the coefficients were computed to cover the important low-frequency part of the spectrum better,

*AR coefficients + Δ coefficients:* 8 first-order derivative approximations (2) were computed and the feature vector was extended to 16 values.

### 6.3. Results

The complete classification was run with all these parameters covering all the scalp electrodes. The results were sorted according to two criteria:

1. overall classification score computed as a weighed average of the little finger, thumb and resting EEG classification scores. This number tells us how good it is possible to discriminate the single types of EEG at the given electrode and for the given subject,
2. false movement rate detection which is a probability measure of a movement detection when the subject is actually resting.

The best results selected according to these criteria for each of the subjects are listed in Table 5. It may be seen that it is possible to distinguish between movement-related and resting EEG and to find an electrode and parameterization which minimizes the possibility of false movement detection for any of the subjects. On the other hand, the classifier was not able to distinguish between the thumb and little finger EEG. Some of the movements are always ignored (less than 10%) recognizing them as resting EEG; however, the thumb—little finger discrimination—failed. Figure 3 with the localization of the electrodes summarizes the best performances from the classification score point of view.

**Figure 4:**
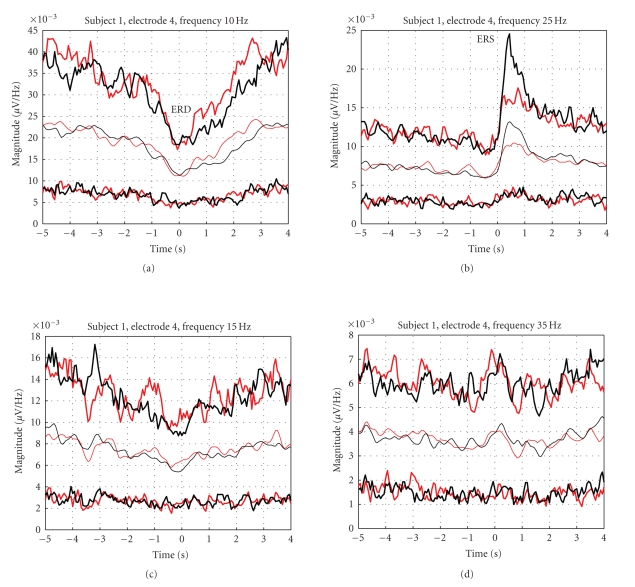
Confidence intervals computed for the selected frequency components (subject 1, electrode 4); see also Figure 1. Black:thumb flexion; red:little-finger flexion; thin lines:average courses of the indicated spectral components; thick lines:boundaries containing 75 % of the real EEG realizations.

Subsequent analysis of the recognized movements showed that—although the mean values of the movement-related EEG spectra are significantly statistically different—the real time courses of the movement-related EEG are heavily buried in the non-movement related activity, see Figure 4.

## 7. CONCLUSIONS, NEXT STEPS

In this work, a detailed finger movement-related EEG statistical analysis and result of classification experiment were presented.

The movement-related EEG was analyzed in an individual way searching for as statistically significant phenomena as possible instead of the commonly used analysis of the strongest EEG component. This approach is in contrast with the method used by [20, 21, 26] and others, where the strongest ERDs and ERSs are found first and their statistical significance is checked afterwards.

We found statistically significant differences between both types of movement-related EEG signals. The differences in the β and μERD parameters were present, although not very important. More interestingly, we discovered significant differences in the βERS courses, their characters being highly individually dependent. These results are promising from the classification point of view. No such results of finger movement-related EEG analysis have been published yet. In addition to this, our analysis covered the whole EEG frequency band (5–30 Hz) and both sensorimotor areas extending the results of [20], where only EEG recorded from C3/C4 positions and only signal powers in 10–12 Hz, 16–20 Hz and 20–24 Hz bands were examined.

Our classification paradigm was only partially successful—we were able to distinguish the movement-related and resting EEG, but the movements were not distinguished from each other. This was attributed to the fact that the movement-related spectral EEG courses are masked by other on-going EEG activities not related to the movement. Thanks to the individual analysis results we believe it will be able to separate and successfully classify both movements with the help of an advanced denoising approach.

In our recent work [37] we showed that it is possible to separate movement-related EEG sources and non-movement related EEG activity with the help of the independent component analysis (ICA). Now we have been working on the integration of an ICA-based denoising procedure into our classification system. This approach should help us to increase the classification score by means of EEG separation into meaningful sources.

Next step will be to combine the developed method with the left/right limb movement recognition to double the number of recognized states—to increase the brain-computer channel data rate.

## Figures and Tables

**Table 1:** The list of the experimental subjects' characteristics. Positive dominance score means that the subject's right hand is more skilled than the lefthand. Right-handed subjects had the average score of 17.2, nonright-handed had 5.2, and left-handed had −10.

Subject	Age	Dominance	Dominant	Little-finger	Thumb	Resting
number	(yrs)	score (–)	hand	epochs	epochs	epochs
1	26	24.18	Right	88	87	90
2	26	5.44	Nonright	55	60	56
3	25	7.92	Nonright	86	86	75
4	25	13.81	Right	94	89	95
5	30	−10.03	Left	66	85	93
6	25	2.29	Nonright	86	85	91
7	18	15.63	Right	83	70	105
8	21	15.92	Right	84	85	132

**Table 2:** The most reactive μERD spectral components' parameters for the single subjects, and contralateral and ipsilateral scalp sides.

Contralateral hemisphere						
Subject	Little-finger			Thumb		
number	Electrode	frequency (Hz)	ERD (%)	Electrode	frequency (Hz)	ERD (%)
1	9	12	−87	9	13	−91
2	1	8	−95	1	8	−95
3	18	12	−87	18	12	−91
4	36	9	−79	30	9	−73
5	18	11	−88	18	12	−89
6	18	13	−93	18	13	−90
7	30	11	−72	30	10	−83
8	36	10	−62	36	10	−66

Ipsilateral hemisphere						
Subject	Little-finger			Thumb		
number	Electrode	frequency (Hz)	ERD (%)	Electrode	frequency (Hz)	ERD (%)

1	24	11	−87	24	11	−91
2	7	8	−89	7	8	−93
3	24	12	−75	24	11	−84
4	33	10	−77	33	9	−72
5	24	11	−85	24	11	−88
6	24	12	−87	24	12	−88
7	24	11	−69	31	10	−75
8	32	11	−66	24	14	−69

**Table 3:** The most reactive βERS spectral components' parameters for the single subjects and contralateral and ipsilateral scalp sides.

Contralateral hemisphere						
Subject	Little-finger			Thumb		
number	Electrode	frequency (Hz)	ERS (%)	Electrode	frequency (Hz)	ERS (%)
1	12	31	262	12	32	222
1	12	29	222	4	26	337
2	17	27	141	37	22	126
3	10	27	260	10	26	231
3	—	—	—	03	10	125
4	8	14	85	27	21	80
5	8	17	132	8	18	95
5	8	28	107	—	—	—
6	10	26	174	1	16	145
6	1	33	131	10	27	143
7	27	21	738	18	21	509
8	9	19	389	9	18	379
8	21	30	164	21	28	236

Ipsilateral hemisphere						
Subject	Little-finger			Thumb		
number	Electrode	frequency (Hz)	ERS (%)	Electrode	frequency (Hz)	ERS (%)

1	12	31	262	4	26	338
1	12	29	221	4	26	338
2	16	28	650	16	35	192
3	15	26	169	16	14	117
4	—	—	—	—	—	—
5	16	32	117	41	17	104
6	14	16	260	14	17	326
7	15	22	383	15	19	664
8	14	19	374	14	21	128
8	—	—	—	23	29	110

**Table 4:** Statistically significant βERS spectral components for the single subjects, summary of the analysis. The location column gives the location of the found components in terms of our electrode numbers, see Figure 3.

Subject number	Movement with stronger βERS	Parameters (time, frequency)	Location (electrode)
1	Thumb	0.5–1 sec, 26-27 Hz	**2**, 3, **4**, **5**, 9, 10, **18**, 19, 24
2		No significant differences	
3	Thumb	0.375–0.875 s, 25–29 Hz	**14**
4		No significant differences	
5	Little	1.250–1.625 s, 16–20 Hz	1, **29**, 31
6	Thumb	0.25–1.125 s, 20–27 Hz	6, 23
7	Thumb	1–1.5 s, 17–23 Hz	5, **7**, **15**, **16**, 24
	Little	1–1.5 s, 17–23 Hz	36, 37, 38, 39
8	Little	1.5–2.0 s, 20–24 Hz	15

**Table 5:** EEG-based movement classification, the best results from the overall classification score and minimalization of false positive movement detection points of view. The meanings of the table fields are as follows: *Subj. no.* = number of the subject, *Scalp loc.* = scalp position which gave the best classification score, *Fingers correct* = weighed classification score for both fingers, correct classification, *Fingers wrg.* = weighed classification score for both fingers, thumb classified as little finger and vice versa, *Fingers ign.* = percentage of finger movements classified as resting EEG—ignored movements, *Fingers false* = false positive detection, percentage of resting EEG realizations classified as movement, *Resting* = classification score of resting EEG, *Total* = overall classification score, weighed average of the single scores, *Parameters* = parameterization used to get the best results.

Results sorted according to overall classification score								
Subj. no.	Scalp loc.	Fingers corr. [%]	Fingers wrg. [%]	Fingers ign. [%]	Fingers false [%]	Resting [%]	Total [%]	Parameters used
1	2	56.1	42.3	1.6	11.4	88.6	67.3	FFT+Δ
2	3	57.8	41.2	1.1	8.5	91.5	68.8	FFT
3	14	51.4	44.4	4.1	1.3	98.7	65.7	AR
4	37	52.9	42.7	4.4	0.0	100.0	68.8	AR
5	29	51.6	48.4	0.0	0.0	100.0	70.0	AR
6	10	53.8	42.8	3.4	28.3	71.7	60.0	FFT+Δ
7	17	57.7	39.4	2.9	1.9	98.1	74.2	AR
8	5	48.8	51.0	0.1	0.4	99.6	70.9	AR

Results sorted according to false positive detections								
Subj. no.	Scalp loc.	Fingers corr. [%]	Fingers wrg. [%]	Fingers ign. [%]	Fingers false [%]	Resting [%]	Total [%]	Parameters used

1	14	35.1	40.2	24.7	0.8	99.2	57.1	AR+Δ
2	1	49.4	46.1	4.5	0.0	100.0	65.8	AR+Δ
3	1	23.4	24.6	52.0	0.3	99.7	46.4	FFT
4	37	52.9	42.7	4.4	0.0	100.0	68.8	AR
5	29	51.6	48.4	0.0	0.0	100.0	70.0	AR
6	1	28.3	34.2	37.5	4.9	94.1	51.2	FFT+Δ
7	18	53.8	45.7	0.5	0.0	100.0	72.7	AR
8	5	48.8	51.0	0.1	0.4	99.6	70.9	AR
